# The League of Mercy

**Published:** 1923-08

**Authors:** 


### THE LEAGUE OF MERCY.

The charming garden at St. James's Palace, which is very rarely used for public functions, was crowded for the garden party which was given to the chief workers of the League of Mercy on Friday, July 13th. Over a thousand acceptances had been received to the invitation to meet the Prince of Wales, the Grand President of the League, and Princess Alice, Countess of Athlone, the Lady President. Consequently, when the Prince came into the garden from Clarence House at four o'clock, the lawns which lie in front of the State apartments were thronged. In the absence abroad of Lord Wolferton, the senior Honorary Secretary, the Prince was received by Sir William Collins and Sir James Harrison, Honorary Secretaries, Sir Edward Wallington, the Honorary Registrar, and Sir Frederick Green, the Honorary Treasurer. It was a delightfully informal entertainment, the Prince moving about the lawns greeting those whom he knew.

The occasion was particularly interesting as this was the first of the annual garden parties of the League which the Grand President and the Lady President have been able to attend together since the Prince of Wales took office.

